# Color-Tunable and Efficient CsPbBr_3_ Photovoltaics Enabled by a Triple-Functional P3HT Modification

**DOI:** 10.3390/ma18194579

**Published:** 2025-10-02

**Authors:** Yanan Zhang, Zhizhe Wang, Dazheng Chen, Tongwanming Zheng, Menglin Yan, Yibing He, Zihao Wang, Weihang Zhang, Chunfu Zhang

**Affiliations:** 1 State Key Laboratory of Wide Bandgap Semiconductor Devices and Integrated Technology, Faculty of Integrated Circuit, Xidian University, Xi’an 710071, China; 24251215323@stu.xidian.edu.cn (Y.Z.); 15346150101@163.com (M.Y.); heyibing0507@163.com (Y.H.); yn1011168573@163.com (Z.W.); whzhang@xidian.edu.cn (W.Z.); 2 Science and Technology on Reliability Physics and Application of Electronic Component Laboratory, China Electronic Product Reliability and Environmental Testing Research Institute, Guangzhou 511370, China; zhizhewang@yeah.net; 3 Guangzhou Wide Bandgap Semiconductor Innovation Center, Guangzhou Institute of Technology, Xidian University, Guangzhou 510555, China; 4 Department of Chemistry, Faculty of Mathematical & Physical Sciences, University College London, London WC1H 9BT, UK; zccatz3@ucl.ac.uk

**Keywords:** CsPbBr_3_, P3HT, light absorption, color-tunable, perovskite solar cells

## Abstract

All inorganic CsPbBr_3_ possesses ideal stability in halide perovskites, but its wide bandgap and relatively poor film quality seriously limit the performance enhancement and possible applications of perovskite solar cells (PSCs). In this work, a triple-functional poly(3-Hexylthiophene) (P3HT) modifier was introduced to realize color-tunable semi-transparent CsPbBr_3_ PSCs. From the optical perspective, the P3HT acted as the assistant photoactive layer, enhanced the light absorption capacity of the CsPbBr_3_ film, and broadened the spectrum response range of devices. In view of the hole transport layer, P3HT modified the energy level matching between the CsPbBr_3_/anode interface and facilitated the hole transport. Simultaneously, the S^−^ in P3HT formed a more stable Pb-S bond with the uncoordinated Pb^2+^ on the surface of CsPbBr_3_ and played the role of a defect passivator. As the P3HT concentration increased from 0 to 15 mg/mL, the color of CsPbBr_3_ devices gradually changed from light yellow to reddish brown. The PSC treated by an optimal P3HT concentration of 10 mg/mL achieved a champion power conversion efficiency (PCE) of 8.71%, with a V_OC_ of 1.30 V and a J_SC_ of 8.54 mA/cm^2^, which are remarkably higher than those of control devices (6.86%, 1.22 V, and 8.21 mA/cm^2^), as well its non-degrading stability and repeatability. Here, the constructed CsPbBr_3_/P3HT heterostructure revealed effective paths for enhancing the photovoltaic performance of CsPbBr_3_ PSCs and boosted their semi-transparent applications in building integrated photovoltaics (BIPVs).

## 1. Introduction

Metal halide perovskite solar cells (PSCs), characterized by their high light absorption coefficient (>10^4^ cm^−1^) [1], tunable bandgap (1.2–2.3 eV) [2], low-cost fabrication, and exceptional PCE (over 27%) [3], have emerged as a highly promising third-generation photovoltaic technology. These advantages enable their applications in photovoltaic power plants, building integrated photovoltaics (BIPVs), and consumer electronics [4,5,6]. Nowadays, glass facades are commonly employed in office buildings, commercial complexes, sunrooms, skylights, and other architectural structures. If conventional glass was replaced with semi-transparent and color-tunable PSCs, not only would the functional benefits of glass facades be preserved but each building could also be transformed into a sustainable source of clean energy. However, the high volatility and thermal instability of organic cations (CH_3_NH_3_^+^, CH(NH_2_)_2_^+^) in hybrid PSCs are still a limitation to realize the high-reliability devices and further industrialization [7,8,9]. To address these stability challenges, researchers have developed all-inorganic PSCs by replacing organic cations with inorganic cesium ions (Cs^+^). Among these, the light-yellow semi-transparent CsPbBr_3_ perovskites with excellent environmental stability and high open-circuit voltage (V_OC_) have attracted increasing attention [10,11,12]. But the intrinsically wide bandgap (~2.3 eV) of CsPbBr_3_ perovskites, manifesting as a narrow visible-light absorption range (≤530 nm), constrains photocurrent generation and PCE for PSCs. This spectral mismatch with solar irradiance substantially hinders their potential commercialization, particularly in BIPVs where tunable coloration and high-power output are critical.

To tackle this issue, significant efforts have been devoted by researchers around the world, including in film treatment, additive strategy, interface modification, and so on. He et al. performed a water vapor treatment on the CsPbBr_3_ film prior to thermal annealing, successfully obtaining a high-quality CsPbBr_3_ film with a purer phase composition and enhanced optical absorbance [13]. Simultaneously, additive strategies including isopropanol or thiourea boosted the grain growth of CsPbBr_3_ perovskites [14,15], and the doping of Sn^2+^ decreased the bandgap and optimized the absorption capacity [16], resulting in improvements in the spectrum response and PCE for CsPbBr_3_ PSCs. Yuan et al. introduced a SnS:ZnS layer into the CsPbBr_3_/HTL interface, and the device achieved an enhanced optical absorption range up to 700 nm and efficient charge transport performance [17]. Meanwhile, Zhao et al. widened the spectral response range to 780 nm by fabricating a CsPbBr_3_/organic J61-ITIC bulk heterojunction and achieved a substantial improvement in the short-circuit current density (J_SC_) [18]. On the other hand, the use of a poly(3-hexylthiophene) (P3HT) hole transport layer enables a better energy level alignment and boosts charge carrier transport [19,20], but its own ability to absorb light (350–640 nm) was overlooked [21]. It is promising to recognize the double identity of P3HT as a light absorber and an interface modifier, which can not only can widen the photoelectric response range and change the color of the PSCs but also coordinate the carrier transport through energy level matching and defect passivation.

In this work, by introducing a thickness-varied P3HT interlayer between the CsPbBr_3_/Ag electrode interfaces, the formed CsPbBr_3_/P3HT heterostructure widened the spectral response range of the devices, reduced the number of recombination centers on the film surface, improved the PCE, and realized the color-tunable CsPbBr_3_ PSCs. In detail, the light absorption ability of the CsPbBr_3_ devices between 350 nm and 640 nm was significantly enhanced; the S^−^ in P3HT forms a more stable Pb-S bond with the uncoordinated Pb^2+^ on the surface of CsPbBr_3_, effectively passivating the Pb^2+^ defects in the lattice.; As the P3HT concentration increased from 0 to 15 mg/mL, the device’s color shifted from light yellow to deep red; the optimized CsPbBr_3_ PSCs achieved a champion PCE of 8.71%, representing a 27% enhancement over the control device (6.86%), with nearly unchanged hydrothermal stability. These results provide a promising color-tunable and efficient photovoltaic technology for the application of perovskites in BIPVs.

## 2. Materials and Methods

The device structure in this work is FTO/TiO_2_/CsPbBr_3_/P3HT/MoO_3_/Ag. The materials and reagents of PbBr_2_ (99.999%, Alfa Aesar, Karlsruhe, Germany), CsBr (99.999%, Alfa Aesar), N,N-Dimethylformamide (DMF, 99.9+%), Isopropanol (IPA, 99.5%, Sigma-Aldrich, Shanghai, China), Poly(3-hexylthiophene) (P3HT, Xi’an Yuri Solar, Xi’an, China), and FTO/glass substrates (TEC-8, 8Ω/sq) were used without further modifications.

To fabricate the PSCs, the dry FTO substrates (ultrasonically cleaned for 15 min by detergent and anhydrous ethanol and nitrogen-dried) were treated in the ultraviolet ozone for 15 min, and then the compact TiO_2_ ETL was deposited using the sol–gel method (spin-coating, 3000 rpm, 30 s) and high-temperature annealing (500 °C, 1 h). The CsPbBr_3_ absorber was prepared via a two-step solution process: a 1.0 M PbBr_2_/DMF precursor solution was first statically spin-coated uniformly onto the TiO_2_ layer (2000 rpm, 30 s) followed by annealing at 90 °C for 30 s, and consequently, the CsBr/H_2_O solution (90 µL, 250 mg/mL) was dynamically spin-coated onto the PbBr_2_ films (2000 rpm, 30 s), and the film was annealed at 250 °C for 5 min. After annealing at 250 °C for 5 min, the polycrystalline CsPbBr_3_ layers were formed. Specifically, the P3HT/CB (Chlorobenzene) solutions with various concentrations (5, 7.5, 10, 12.5, 15 mg/mL) were dynamically spin-coated onto perovskites at 1500 rpm and 4500 rpm for 5 s and 45 s, respectively. The MoO_3_ buffer layer and ultrathin transparent silver (Ag) electrode were evaporated, and finally the semi-transparent CsPbBr_3_-PSCs were obtained.

The optical and micro-structure properties of CsPbBr_3_ samples were investigated by the UV-vis spectrophotometer, photoluminescence (PL), scanning electron microscope (SEM), X-ray photoelectron spectroscopy (XPS), and ultraviolet photoelectron spectroscopy (UPS). To evaluate the photovoltaic performance, the current density–voltage (J-V) and external quantum efficiency (EQE) curves were measured by Keithley 2400 source meter, AM 1.5 G, and SCS10-X150 quantum efficiency tester. And the transient photocurrent (TPC), photovoltage (TPV) decay curves, electrochemical impedance spectroscopy (EIS), and M-S plots were tested to study the carrier transport behavior. More details of these measurements can be found in our previous works [14].

## 3. Results and Discussion

**Figure 1:**
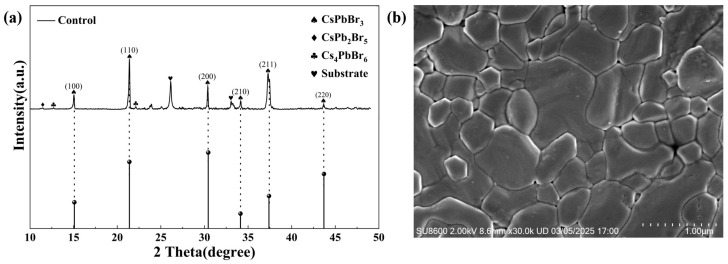
(**a**) XRD spectrum and (**b**) SEM image of the control CsPbBr_3_ film on FTO/TiO_2_ substrate.

The crystal structure of CsPbBr_3_ is a cubic phase, which consists of a [PbBr_6_]^4−^octahedra, forming a three-dimensional network through the shared vertices, with Cs^+^ ions occupying the octahedral gaps. During the process of preparation, in addition to the formation of the 3D CsPbBr_3_ phase with better light absorption, phases with poor light absorption properties, such as the 2D CsPb_2_Br_5_ phase and the 0D Cs_4_PbBr_6_ phase, were also formed, which seriously affected device performance. It is noted in Figure 1a that the pristine CsPbBr_3_ films prepared in this study exhibited typical CsPbBr_3_ diffraction peaks with a small amount of impurity phases. Figure 1b showed the SEM image of the control CsPbBr_3_ film; a smooth and compact surface can be observed as well as some defects (like pin holes) in the prepared film. These defects may further transform into the recombination centers of carriers, thereby adversely affecting device performance. Also, the relatively large energy barrier between the CsPbBr_3_ and electrode would hinder the efficient extraction and transport of carriers [22]. Moreover, a wide bandgap of 2.3 eV fundamentally limits the light absorption capacity of the CsPbBr_3_ perovskite. Here, the triple-functional P3HT interlayer was employed to address the above problems and improve the performance of CsPbBr_3_ PSCs.

**Figure 2:**
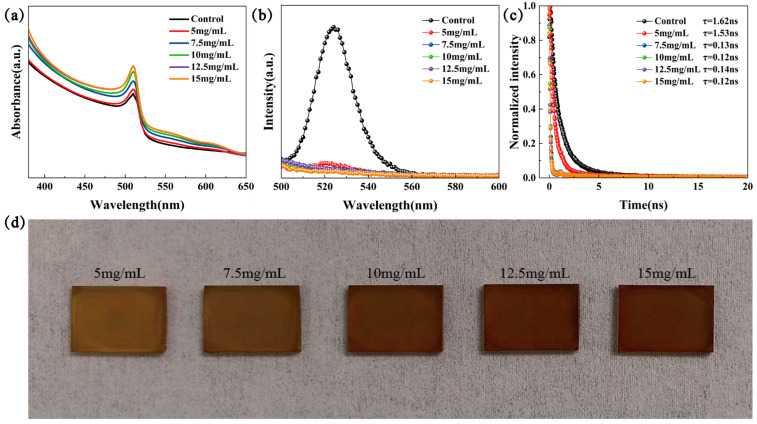
(**a**) UV-vis, (**b**) PL, (**c**) TRPL, and (**d**) photos of the FTO/TiO_2_/CsPbBr_3_/P3HT samples with various concentrations of P3HT solution.

Firstly, we set up the concentration gradient (0, 5, 7.5, 10, 12.5, and 15 mg/mL) of P3HT solutions to systematically examine the effect of the P3HT thickness on the efficiency and light absorption properties of CsPbBr_3_ devices. Figure 2a presented the UV-Vis test results of the six samples with different conditions, and all samples spin-coated with P3HT exhibit an enhanced light absorption capacity in the wavelength range from 350 nm to 640 nm. Here, the absorption cutoff edge of CsPbBr_3_ films did not change, and the improvement within the 530 nm to 640 nm range can be attributed to the contribution of the P3HT absorber [21]. From the PL spectra in Figure 2b, the PL feature peaks of FTO/TiO_2_/CsPbBr_3_/P3HT samples were quenched rapidly sharply compared with the control sample, which demonstrates that the introduction of the P3HT significantly enhances the carrier extraction efficiency. It is clear from the TRPL test and fit results in Figure 2c that the carrier decay time of the samples with P3HT concentrations ranging from 7.5 to 15 mg/mL decreased rapidly, confirming their efficacy as hole transport layers. In contrast, the longer decay time at 5 mg/mL may be attributed to the hole transport layer of P3HT, with a concentration that is too low discontinuous and, therefore, cannot function effectively [20]. Figure 2d shows the gradual evolution of the CsPbBr_3_/P3HT sample’s color from light yellow to reddish brown with the P3HT concentrations increased from 0 to 15 mg/mL. This color-tunable property is expected in the possible applications in BIPVs.

**Figure 3:**
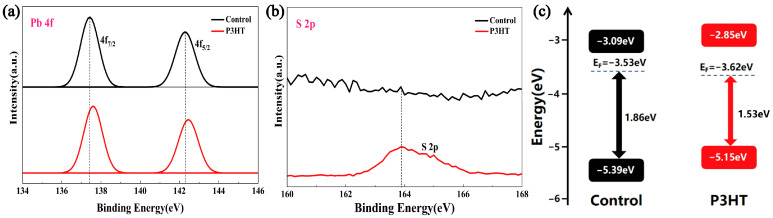
(**a**) Pb 4f, (**b**) S 2p XPS spectra, and (**c**) energy band structure calculated from the UPS spectra for the control and P3HT-modified samples (10 mg/mL). The XPS spectra of C 1s, Cs 3d, and Br 3d can be found in Figure S2.

Next, we investigated the effect of the P3HT treatment on the surface elemental valence states of CsPbBr_3_ films with the XPS test, and the results show that the binding energies of Cs and Br remained basically unchanged after the P3HT treatment, while the Pb 4f peak shifted toward a higher binding energy by 0.2–0.25 eV (Figure 3a), which should be attributed to the strong bonding between S and Pb, and the S 2p peak appeared at 163.9 eV (Figure 3b), confirming the sulfidation of the perovskite. The Tauc plot (Figure S1a,b) shows that the bandgap (2.3 eV) of the CsPbBr_3_ films did not change before or after the P3HT treatment, indicating that P3HT did not change the lattice structure. Then, we performed the analysis of the energy band structure through UPS characterizations, as shown in Figure S1 and Figure 3c; the CsPbBr_3_/P3HT sample obtained a shallower valence band bottom of −5.15 eV compared to that of the control CsPbBr_3_ sample (−5.39 eV), the corresponding values of work functions increase from 3.53 eV to 3.62 eV, and the surface energy bands at the contact interface of CsPbBr_3_/P3HT tend to bend upwards. Consequently, the constructed CsPbBr_3_/P3HT heterostructure provides a favorable energy level alignment at the interface, enhancing the hole extraction and suppressing the carrier recombination near the anode. The corresponding photovoltaic performance of PSCs is discussed in the following paragraphs.

**Figure 4:**
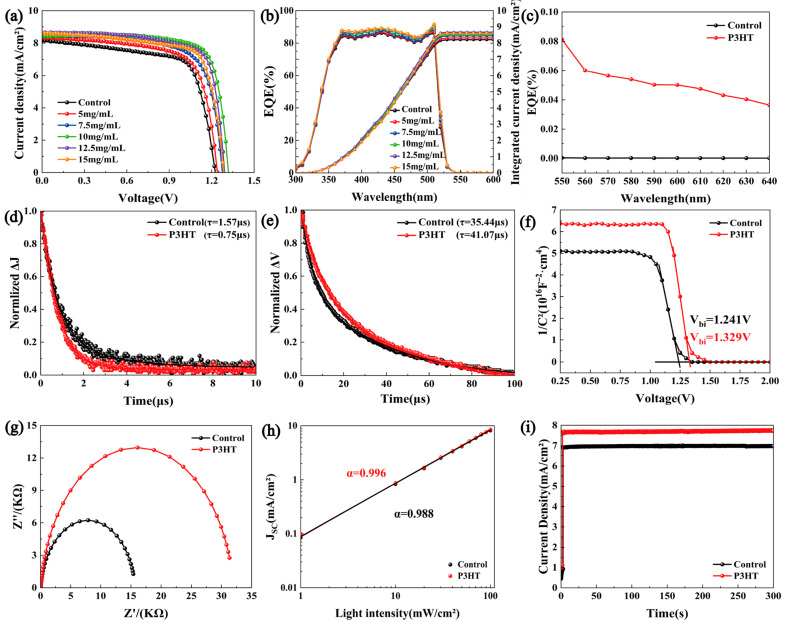
(**a**) J-V, (**b**) EQE and integrated current densities, (**c**) IPCE spectra of P3HT processing devices (550 nm–640 nm), (**d**) TPC, (**e**) TPV, (**f**) C-V, (**g**) EIS, (**h**) light intensity-dependent PCE curves of control and P3HT treatment films, and (**i**) steady-state current outputs.

J-V curves in regard to the structure of FTO/TiO_2_/CsPbBr_3_/P3HT/MoO_3_/Ag and different concentrations of the P3HT solution are displayed in Figure 4a, and the photovoltaic parameters of all samples are listed in Table 1. The control device (without a P3HT modifier) showed a PCE of 6.86%, a V_OC_ of 1.22 V, a J_SC_ of 8.21 mA/cm^2^, and an FF of 68.44%. The device with a P3HT concentration of 10 mg/mL exhibited the best photovoltaic performance, achieving a champion PCE of 8.71%, with V_OC_, J_SC_, and FF values of 1.30 V, 8.54 mA/cm^2^, and 78.42%, respectively. This result can be further verified by the statistical distribution of the PCE, V_OC_, J_SC_, and FF in Figure S3. Also, the P3HT-modified device exhibited a lower leakage current in the dark (see Figure S4). The overall improvement of all parameters benefited from the enhanced absorption ability, improved hole extraction and transportation, and suppressed carrier recombined in the PSCs, which are in line with the above characterized results from optical and electrical perspectives. For the PSCs with a lower P3HT concentration of 5 mg/mL, the relatively poor performance can be explained by the P3HT being unable to completely cover the CsPbBr_3_ film. With the concentration increased to 10 mg/mL, the P3HT could fully cover the CsPbBr_3_ surface, which is proven by the SEM image shown in Figure S5, and effectively play the role of the triple-function modifier. However, when the concentration of P3HT was further increased, although larger J_SC_ values can be obtained, the PCE of the device started to decrease. The larger series resistance of PSCs induced by the thicker P3HT layer may be responsible for this tendency [23,24]. Additionally, an excessively thick P3HT layer will generate a mass of electron–hole pairs, which will increase the possibility of carrier’s recombination during the hole transport process, thus reducing the V_OC_, FF, and the PCE of the solar cell. On the other hand, Figure 4b shows the EQE curves and integrated current results of CsPbBr_3_ PSCs. It is clear that the devices with the P3HT modification showed a higher EQE over the spectra response range (300 nm–540 nm) with the increased P3HT concentration, which agrees with the light absorption results in Figure 2a. And the values of the integrated current density are very close to the J_SC_ and also demonstrate the same change tendency with the various concentrations of P3HT. It is worth noting that the noticeable EQE between 550 nm and 640 nm can be found in Figure 4c for the P3HT-modified CsPbBr_3_ PSCs, which suggested that the P3HT could broaden the wavelength range of the photo-electrical conversion of PSCs.

TPC and TPV decay curve tests were conducted to further investigate the carrier transport behaviors. It is known that the TPC reflects the carrier extraction and transport properties, while the TPV can reveal the recombination characteristics of the carriers within the solar cells [25,26,27]. In Figure 4d, the fitted photocurrent decay time of the control devices was 1.57 μs, whereas the P3HT-treated devices exhibited a significantly faster photocurrent decay time of 0.75 μs, indicating a more efficient charge extraction process in the corresponding PSCs. Figure 4e presents the TPV test results. Compared to the photovoltage decay time of 35.44 μs for the control one, the P3HT-modified device showed a markedly slower photovoltage decay time of 41.07 μs, suggesting that the introduction of P3HT effectively suppressed the carrier recombination and increased the carrier lifetime. Therefore, the efficient carrier extraction and transportation supported the superior photovoltaic performance of CsPbBr_3_/P3HT devices.

**Figure 5:**
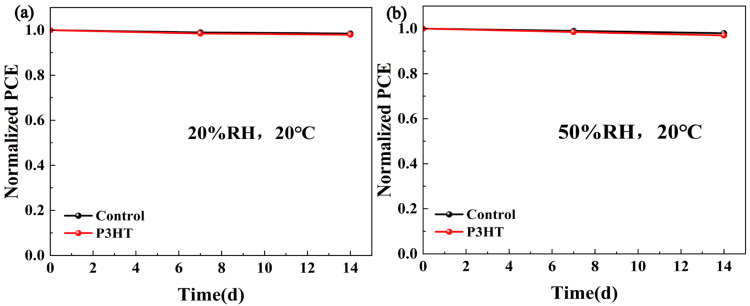
Storage stability of unencapsulated devices: (**a**) 20% RH, 20 °C and (**b**) 50% RH, 20 °C.

The built-in electric field (V_bi_) of cells was extracted from the C-V test results in Figure 4f, and remarkably the V_bi_ value of devices increased from 1.241 V to 1.329 V after the P3HT modification. The higher built-in voltage facilitates carrier transport and forms a wider depletion region, thereby suppressing the carrier recombination [28,29]. Additionally, the enhanced V_bi_ explains the higher V_OC_ in the corresponding CsPbBr_3_/P3HT cells. The EIS test results are shown in Figure 4g. A larger arc radius indicates a higher recombination resistance (R_rec_), suggesting that the non-radiative recombination was suppressed in the P3HT-modified PSCs. Figure 4h displays the light intensity-dependent J_SC_ for the solar cell. The α value closer to one suggests a lower bimolecular recombination in the PSCs. The α value increased from 0.988 in the original devices to 0.996 in the P3HT-treated CsPbBr_3_ PSCs, indicating suppressed bimolecular recombination and enhanced charge carrier separation. The steady output curves at the maximum power point in Figure 4i and both the control and P3HT-modified devices showed the stable photocurrent output ability, and the optimized device obtained an increased steady current density from 6.8 mA/cm^2^ to 7.6 mA/cm^2^, which demonstrated the excellent stability of PSCs under the continuous illumination. In addition, we conducted a comparative stability study of the PSCs with and without the P3HT modification under various environmental conditions. As shown in Figure 5a, when the unencapsulated devices were stored in a drying cabinet (20%RH, 20 °C) for two weeks, all the devices maintained approximately 98% of their original PCEs. Figure 5b shows the normalized PCE decay curves of the samples placed in a laboratory environment (50%RH, 20 °C). The results demonstrated that both the control and P3HT-modified devices retained about 97% of their initial efficiency. The results indicate that the stability of the devices modified with P3HT was not compromised.

## 4. Conclusions

In summary, the color-tunable semi-transparent CsPbBr_3_ PSCs were fabricated by introducing a triple-functional P3HT modifier with various concentrations. The film characterization results showed that the P3HT could enhance the light absorption capacity of the CsPbBr_3_ film, broaden the spectrum response range of CsPbBr_3_ devices, passivate the defects at the CsPbBr_3_ surface, and align the energy levels between the CsPbBr_3_ and anodes. When the P3HT concentration increased from 0 to 15 mg/mL, the color of CsPbBr_3_ devices gradually changed from light yellow to reddish brown. The champion PCE of 8.71% was achieved at an optimal P3HT concentration of 10 mg/mL, as well the improved V_OC_ of 1.30 V and J_SC_ of 8.54 mA/cm^2^. The devices also presented a low leakage current in the dark, great repeatability, and excellent stability under the continuous illumination and air environment. The obviously enhanced carrier extraction and transportation and suppressed non-radiative recombination were responsible for the superior photovoltaic performance of PSCs. Here, the constructed CsPbBr_3_/P3HT heterostructure not only explored an effective strategy to address the PCE limits of wide-bandgap CsPbBr_3_ PSCs but also paved the way for semi-transparent applications of CsPbBr_3_ perovskites in BIPVs.

## Figures and Tables

**Table 1:** Photovoltaic parameters after modification with P3HT at different concentrations.

Condition (mg/mL)	PCE (%)	FF (%)	V_OC_ (V)	J_SC_ (mA/cm^2^)
0 (control)	6.86	68.44	1.22	8.21
5	7.16	69.43	1.24	8.32
7.5	7.62	71.53	1.26	8.45
10	8.71	78.42	1.30	8.54
12.5	8.39	75.64	1.28	8.67
15	8.07	73.35	1.28	8.60

## Data Availability

The original contributions presented in this study are included in the article/Supplementary Material. Further inquiries can be directed to the corresponding authors.

## Supplementary Materials

The following supporting information can be downloaded at https://www.mdpi.com/article/10.3390/ma18194579/s1. Figure S1: (a,b) Tauc plot, (c,d) ultraviolet photoelectron spectroscopy (UPS) images of control and P3HT-modified films (10 mg/mL); Figure S2: (a) C 1s, (b) Cs 3d, (c) Br 3d XPS spectra of Control and P3HT-modified films (10 mg/mL); Figure S3: Statistics photovoltaic parameters of control and P3HT-modified devices (10 mg/mL); Figure S4: Dark J-V curves of Control and P3HT-modified devices (10 mg/mL); Figure S5: SEM image of P3HT-modified film; Figure S6: Partial enlargement of EQE spectra from 515 nm to 535 nm in Figure 4b; Figure S7: (a) PL, (b) TRPL of control and P3HT (10 mg/mL) modified CsPbBr_3_ samples without ETL; Figure S8: Light transmission of control and P3HT (10 mg/mL) modified CsPbBr_3_ films; Figure S9: The XPS full-spectrum of pristine CsPbBr_3_ film. Tables S1 and S2: TRPL fitting parameters before and after P3HT treatment for test samples with and without ETL; Tables S3 and S4: TPC and TPV fitting parameters of PSCs before and after P3HT treatment; and Table S5: Comparation with recent literature of semi-transparent CsPbBr_3_ PSCs.
