# Supplementary material for: Color-Tunable and Efficient CsPbBr3 Photovoltaics Enabled by a Triple-Functional P3HT Modification
Source: Materials (Basel). 2025 Oct 2;18(19):4579. doi: 10.3390/ma18194579 (PMC12526452; doi:10.3390/ma18194579)
Supplement: Supplementary file 1 [file materials-18-04579-s001.zip › materials-3859193-supplementary.pdf]

materials

Supporting Information

Color-tunable and Efficient $CsPbBr_3$ Photovoltaics Enabled by the Triple-functional P3HT Modification

Yanan Zhang [a], Zhizhe Wang [b], Dazheng Chen *[,a,d], Tongwanming Zheng [c], Menglin Yan [a,d], Yibing He [a], ZihaoWang [a], Weihang Zhang [a,d] and Chunfu Zhang *[,a]

a State Key Laboratory of Wide Bandgap Semiconductor Devices and Integrated Technology, Faculty of Integrated Circuit, Xidian University, Xi'an, Shaanxi, 710071, China
b Science and Technology on Reliability Physics and Application of Electronic Component Laboratory, China electronic product reliability and environmental testing research institute, Guangzhou, 511370, China
c University College London, London, WC1H 9BT, United Kingdom
d Guangzhou Wide Bandgap Semiconductor Innovation Center, Guangzhou Institute of Technology, Xidian University, Guangzhou 510555, China
*Corresponding author. Dazheng Chen (dzchen@xidian.edu.cn) and Chunfu Zhang (cfzhang@xidian.edu.cn)

1. Fitting Procedure and Formulas for UPS Spectra

The original UPS spectra are shown in Figures S1c, d. The UPS results were obtained after calibrating the Fermi level using the intrinsic Ag lattice. The work function (W_F) of the material can be calculated using Equation (1):

$$W_F=21.22-E_{cut\_off} \tag{1}$$

where E_{cut_off} is the secondary electron cutoff edge, obtained from the higher binding energy side of the UPS spectrum. The valence band maximum (E_{VBM}) of the material can be calculated using Equation (2):

$$E_{VBM}=-(W_F+(E_F-E_{VBM})) \tag{2}$$

where E_F–E_{VBM} represents the energy difference between the valence band maximum and the Fermi level, derived from the lower binding energy side of the UPS curve.

For the films before and after P3HT modification, the values of E_{cut_off} were 17.69eV and 17.60eV, respectively, and the values of E_F–E_{VBM} were 1.86eV and 1.53eV, respectively. Using Equation (1), the work functions of the materials before and after treatment were calculated to be 3.53eV and 3.62eV, respectively. Applying Equation (2), the E_{VBM} values of the films were determined to be -5.39eV and -5.15eV, respectively. Based on the band gap values of the films before and after treatment, the conduction band minimum (E_{CBM}) positions were further derived as -3.09eV and -2.85eV, respectively. A schematic diagram of the energy band structures of the films before and after treatment is presented in Figure 3c.

2. Experimental text parameters

EIS：Init E (V) = 1；High Frequency (kHz) = 100；Low Frequency (Hz) = 100；Amplitude (V) = 0.005；Quiet Time (sec) = 2；

TPC：Laser wavelength: 532nm (green light)；Internal impedance: 50 Ω

TPV：Laser wavelength: 405nm (violet light)；Amplitude: 5 V；Bias voltage: 2.5 V Frequency: 500 Hz；Waveform: Square wave；Internal impedance: 1 MΩ

3. Results

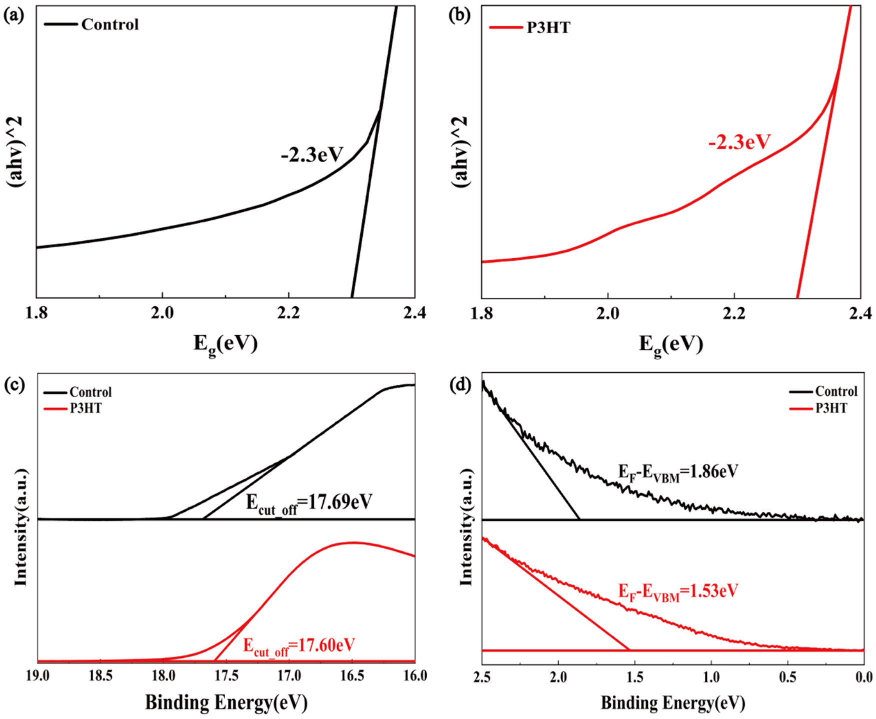

Figure S1. a, b) Tauc plot, (c,d) ultraviolet photoelectron spectroscopy (UPS) images of control and P3HT modified films (10 mg/mL).

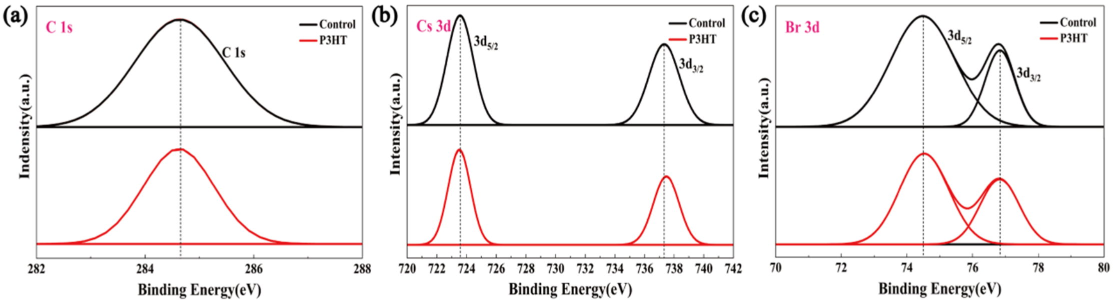

Figure S2. (a)C 1s, (b)Cs 3d, (c)Br 3d XPS spectra of control and P3HT modified films (10 mg/ml).

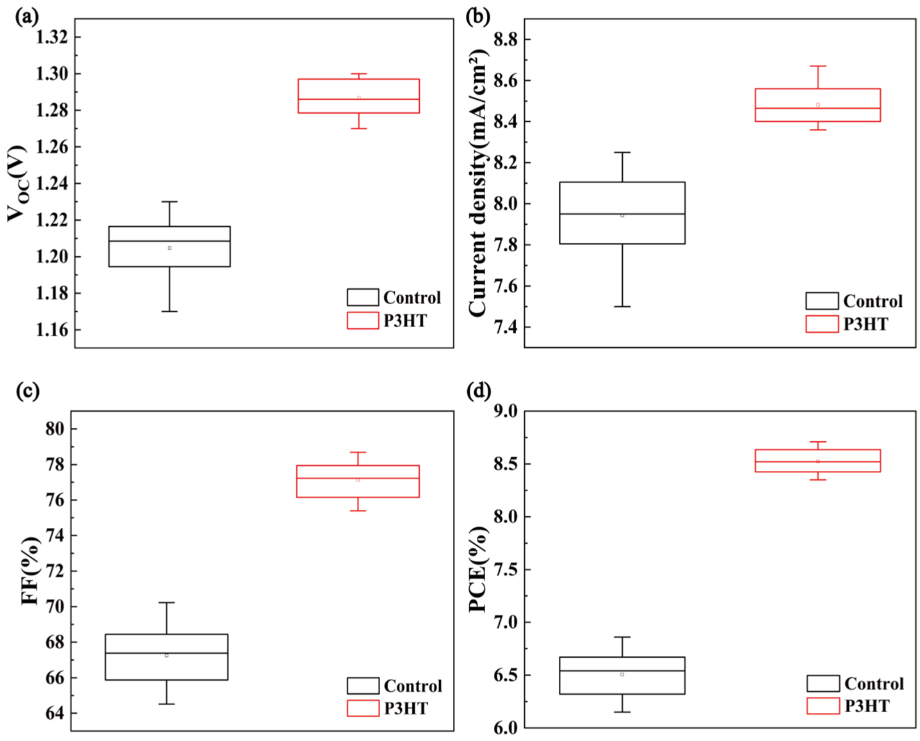

Figure S3. Statistics photovoltaic parameters of control and P3HT modified devices (10 mg/ml).

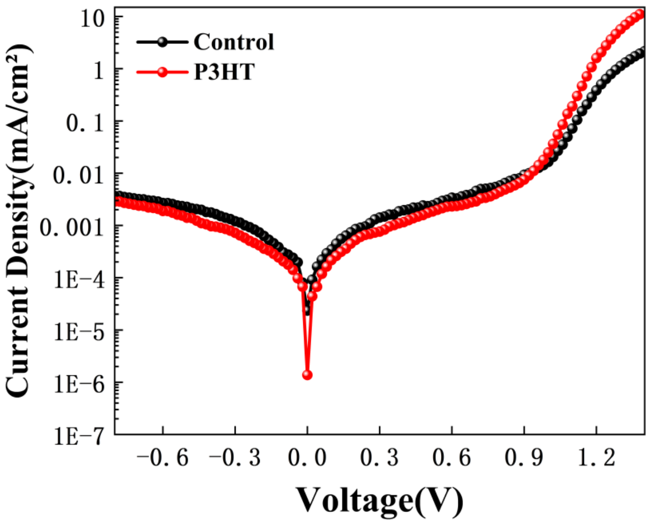

Figure S4. Dark J-V curves of control and P3HT modified devices (10 mg/ml).

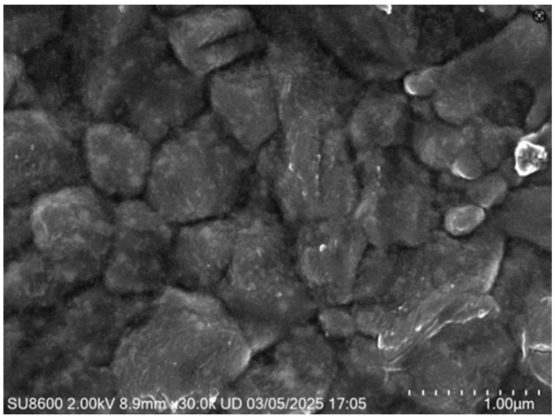

Figure S5. SEM image of P3HT modified film.

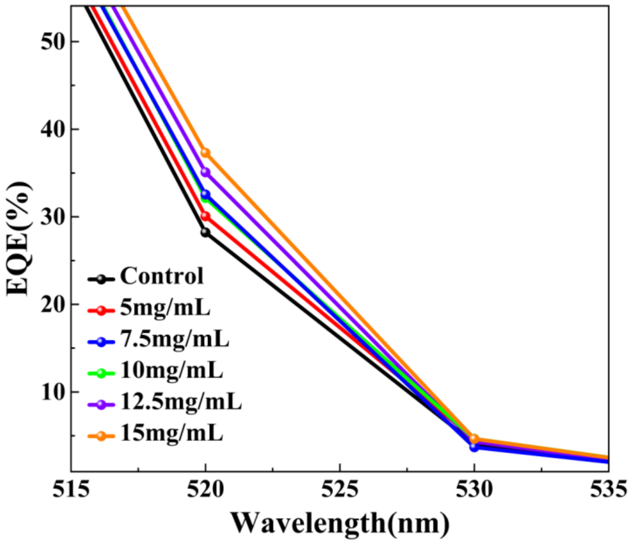

Figure S6. Partial enlargement of EQE spectra from 515 nm to 535 nm in Fig. 4(b).

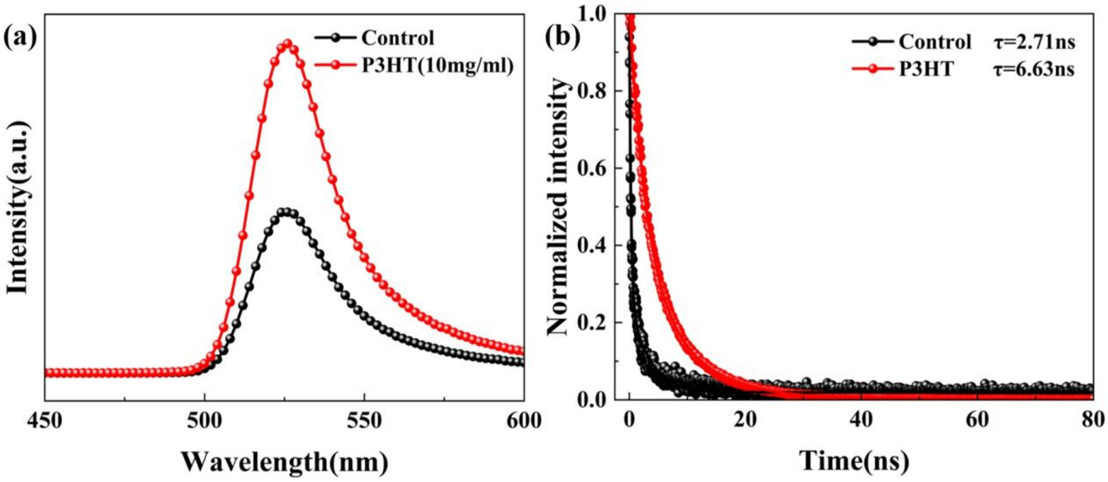

Figure S7. (a)PL, (b)TRPL of control and P3HT (10 mg/ml) modified $CsPbBr_3$ samples without ETL.

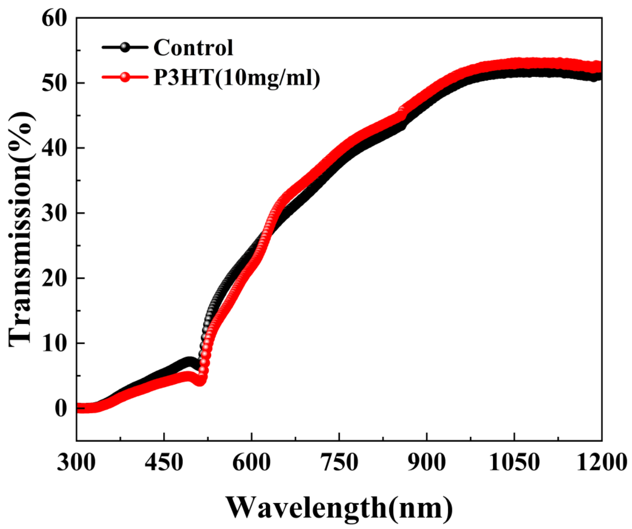

Figure S8 Light transmission of control and P3HT (10 mg/ml) modified $CsPbBr_3$ films.

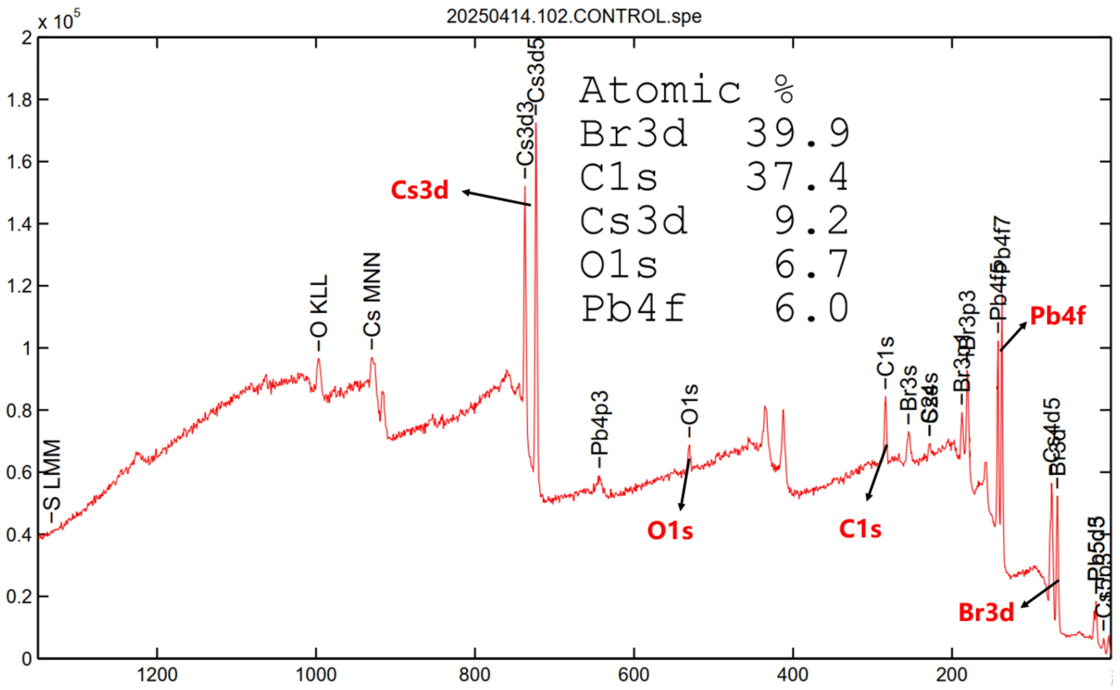

Figure S9. The XPS full-spectrum of pristine $CsPbBr_3$ film.

Table S1. TRPL fitting parameters before and after P3HT treatment for test samples with ETL.

Condition(mg/ml)	A_1	τ_1(ns)	A_2	τ_2(ns)	τ_{ave}(ns)
0(Control)	0.79	0.78	0.18	2.69	1.62
5	0.88	0.68	0.13	2.89	1.53
7.5	0.78	0.09	0.23	0.19	0.13
10	0.8	0.11	0.23	0.14	0.12
12.5	0.81	0.11	0.21	0.21	0.14
15	0.8	0.08	0.21	0.18	0.12

Table S2. TRPL fitting parameters before and after P3HT treatment for test samples without ETL.

Condition	A_1	τ_1(ns)	A_2	τ_2(ns)	τ_{ave}(ns)

Control	0.73	0.29	0.24	3.35	2.71
P3HT	0.63	2.78	0.39	8.63	6.63

Table S3. TPC fitting parameters of PSCs before and after P3HT treatment.

Condition	A_1	τ_1(μs)	A_2	τ_2(μs)	τ_{ave}(μs)
Control	0.72	0.68	0.25	2.32	1.57
P3HT	0.49	0.75	0.52	0.75	0.75

Table S4. TPV fitting parameters of PSCs before and after P3HT treatment.

Condition	A_1	τ_1(μs)	A_2	τ_2(μs)	τ_{ave}(μs)
Control	0.47	5.23	0.57	38.8	35.44
P3HT	0.43	9.04	0.62	45.48	41.07

Table S5. Comparation with recent literature of semi-transparent $CsPbBr_3$ PSCs.

Device Structure	PCE (%)	Reference
FTO/SnO_2/$CsPbBr_3$/ NiO_x/ITO	7.28	[1]
FTO/c-TiO_2/SnO_2/$CsPbBr_3$/ PTAA/ITO	6.11	[2]
FTO/SnO_2/$CsPbBr_3$/P3HT/NiO_x/ITO	7.10	[3]
FTO/c-TiO_2/mp-TiO_2/$CsPbBr_3$/SWNT	8.68	[4]
ITO/SnO_2/$CsPbBr_3$/Spiro-OMeTAD/MoO_3/Au	8.35	[5]
ITO-PEN/ZnO/$CsPbBr_3$/Cu_2O/Spiro-OMeTAD/Au	5.67	[6]
FTO/c-TiO_2/$CsPbBr_3$/ P3HT/MoO_3/Ag	**8.71**	**This work**

Reference

[1] Jiang X, Geng C, Yu X, et al. Doping with KBr to achieve high-performance CsPbBr3 semitransparent perovskite solar cells. ACS Applied Materials & Interfaces, 2024, 16(15): 19039-19047.

[2] Barichello J, Paci B, Moras P, et al. Exploiting the impact of Ionic Liquids and light exposure on performance of fully inorganic CsPbBr3 semi-transparent perovskite solar cells. Solar Energy, 2025, 287: 113237.

[3] Pan J, Chen J, Duan B, et al. Electron-beam-evaporated NiOx for efficient and stable semi-transparent perovskite solar cells and modules. Journal of Materials Chemistry A, 2025, 13(2): 1230-1239.

[4] Daiguji H, Takano H, Watanabe I, et al. All-solution-processed inorganic CsPbBr3 solar cells and their bifacial-irradiation functions. Sustainable Energy & Fuels, 2024, 8(23): 5366-5378.

[5] Xin Y, Zou S, Jin J, et al. Constructing Bionic Perovskite Smart Photovoltaic Windows with Switchable Colors and High Cycling Stability. Small, 2024, 20(52): 2406986.

[6] Tan Y, Xiao B, Xu P, et al. Improving the photovoltaic performance of flexible solar cells with semitransparent inorganic perovskite active layers by interface engineering. ACS Applied Materials & Interfaces, 2021, 13(17): 20034-20042.

Disclaimer/Publisher's Note: The statements, opinions and data contained in all publications are solely those of the individual author(s) and contributor(s) and not of MDPI and/or the editor(s). MDPI and/or the editor(s) disclaim responsibility for any injury to people or property resulting from any ideas, methods, instructions or products referred to in the content.
